# LP1 and LP2: Dual-Target MOPr/DOPr Ligands as Drug Candidates for Persistent Pain Relief

**DOI:** 10.3390/molecules26144168

**Published:** 2021-07-08

**Authors:** Lorella Pasquinucci, Carmela Parenti, Zafiroula Georgoussi, Lorena Reina, Emilia Tomarchio, Rita Turnaturi

**Affiliations:** 1 Department of Drug and Health Sciences, Medicinal Chemistry Section, University of Catania, Viale A. Doria 6, 95125 Catania, Italy; 2 Department of Drug and Health Sciences, Pharmacology and Toxicology Section, University of Catania, Viale A. Doria 6, 95125 Catania, Italy; cparenti@unict.it; 3 Laboratory of Cellular Signaling and Molecular Pharmacology, Institute of Biosciences and Applications, National Centre for Scientific Research “Demokritos” Ag. Paraskevi-Attikis, 15310 Athens, Greece; iro@bio.demokritos.gr; 4 Postgraduate School of Clinical Pharmacology, Toxicology University of Catania, via S. Sofia n. 97, 95100 Catania, Italy; uni303423@studium.unict.it; 5 Postgraduate School of Anesthesiology and Intensive Care, University of Milan, Via Francesco Sforza, 35, 20122 Milan, Italy; Emilia.tomarchio@unimi.it

**Keywords:** benzomorphan, mu opioid receptor, delta opioid receptor, multitarget ligands, biased agonism, pain

## Abstract

Although persistent pain is estimated to affect about 20% of the adult population, current treatments have poor results. Polypharmacology, which is the administration of more than one drug targeting on two or more different sites of action, represents a prominent therapeutic approach for the clinical management of persistent pain. Thus, in the drug discovery process the “one-molecule-multiple targets” strategy nowadays is highly recognized. Indeed, multitarget ligands displaying a better antinociceptive activity with fewer side effects, combined with favorable pharmacokinetic and pharmacodynamic characteristics, have already been shown. Multitarget ligands possessing non-opioid/opioid and opioid/opioid mechanisms of action are considered as potential drug candidates for the management of various pain conditions. In particular, dual-target MOPr (mu opioid peptide receptor)/DOPr (delta opioid peptide receptor) ligands exhibit an improved antinociceptive profile associated with a reduced tolerance-inducing capability. The benzomorphan-based compounds LP1 and LP2 belong to this class of dual-target MOPr/DOPr ligands. In the present manuscript, the structure–activity relationships and the pharmacological fingerprint of LP1 and LP2 compounds as suitable drug candidates for persistent pain relief is described.

## 1. Introduction

In pain transmission the multitude and complexity of involved neuronal mechanisms provide several possible targets for pharmacological intervention [1]. Thus, different antinociceptive drugs with distinct mechanisms and sites of action can provide pain relief.

**Figure 1:**
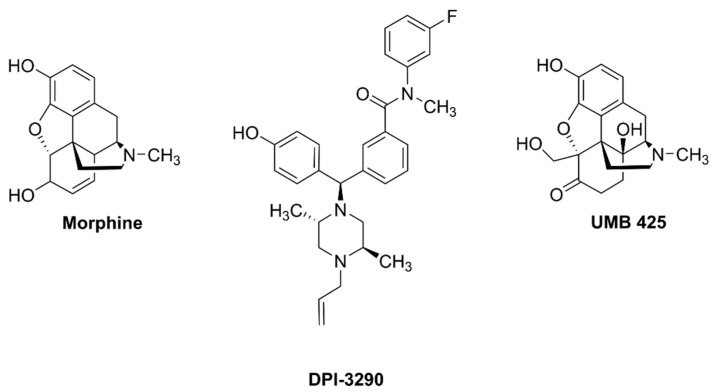
Structures of morphine, DPI-3290 and UMB 425.

For instance, nonsteroidal anti-inflammatory drugs (NSAIDs) are recommended for acute and chronic musculoskeletal and post-surgical pain management [2,3]. Neuropathic pain and post-herpetic neuralgia (PHN) relief may be evoked with local anesthetics such as the well-known lidocaine patch [4,5]. Additionally, ligands acting on the α2-δ subunit of voltage dependent calcium channels are often used in the management of neuropathic pain, such as diabetic neuropathy and PHN [6,7]. Other analgesic/adjuvant agents used to treat persistent pain states are represented by monoamine reuptake inhibitors such as TCAs (e.g., amitriptyline, clomipramine, desipramine and imipramine), and serotonin-noradrenaline reuptake inhibitors (SNRIs) (e.g., duloxetine, milnacipran and venlafaxine) [8,9,10]. However, opioid analgesics continue to be first-line agents for the relief of acute nociceptive pain and chronic cancer pain, and most of them are MOPr (mu opioid peptide receptor) agonists, such as morphine (Figure 1) [11].

Although MOPr agonists represent the first treatment option for nociceptive pain management, their use in long-term treatment of persistent pain states is limited by side effects [12,13]. It has been proved that antinociception and side effects are both elicited by MOPr activation. Building upon these observations and considering that a single-target antinociceptive agent may not provide optimal antinociception, multidrug analgesic approaches or polypharmacology have been introduced into clinical practice for the treatment of both acute and persistent pain [14]. Various clinical trials, performed on patients that underwent minor surgery, revealed additive or synergistic analgesic effects by the combination of NSAIDs with systemic opioids [15], revealing the ability of NSAIDs to reduce the dose of opioids, with subsequent reduction of opioid-induced side effects. Similarly, in patients with persistent pain conditions such as neuropathic pain and PHN, an improved analgesic efficacy was reported for a multidrug regimen consisting of morphine or oxycodone with pregabalin [16], gabapentin with nortriptyline [17]. Moreover, the co-administration of two opioid drugs improved the analgesia MOPr-elicited by reducing the incidence of side effects [18,19]. The co-administration of oxycodone and low-dose naloxone (a non-selective opioid antagonist) decreased the development of constipation while maintaining a valuable analgesic effect, thus improving the safety and tolerability of the treatment [20,21]. However, a polypharmacology approach provides risks related to different pharmacokinetic/pharmacodynamic relationships between co-administered drugs that could lead to unpredictable biological variability [22,23].

In this perspective the past medicinal chemistry paradigm “one-target, one-disease” has been reconverted into “one-molecule, multiple targets” through the development of multitarget ligands [24,25]. Multitarget ligand—a single antinociceptive drug able to act on different receptor sites, also with different mechanisms of action—could represent a valid approach for pain management. In comparison with the multidrug therapy approach, these compounds display more predictable pharmacokinetic and pharmacodynamic properties with an improved patient compliance and lowered risk of drug–drug interactions [26].

Investigations in recent years showed that ligands targeting different opioid receptor types simultaneously could represent suitable candidates for the treatment of persistent pain [27]. First, favorable interactions between MOPr and DOPr were demonstrated by numerous pharmacological studies. An improved side effects profile associated with an increased antinociceptive effect resulted from the co-administration of an MOPr agonist with a DOPr antagonist [28]. Also, the co-administration of a MOPr agonist and a DOPr agonist could produce antinociception with reduced side effects [29,30]. Later, dual-target ligands with MOPr/DOPr (delta opioid peptide receptor) agonist or MOPr agonist/DOPr antagonist activity also showed an improved antinociception and a low propensity to develop side effects. For instance, DPI-3290 [31] (Figure 1), with a balanced MOPr/DOPr affinity profile and the ability to inhibit the contractility of electrically stimulated guinea pig ileum (GPI) and mouse vas deferens (MVD), produced a significant antinociceptive effect, totally and partially blocked by naloxone (NLX) and naltrindole (NTI), respectively. The MOPr agonist/DOPr antagonist UMB 425 [32] (Figure 1), subcutaneously (s.c.) administered, showed a significant antinociceptive effect. Moreover, the development of bifunctional MOPr agonist/DOPr antagonist peptidomimetics was also described [33].

This review aims to point out the progress in the search for opioid multitarget ligands for persistent pain relief, with a focus on the dual-target MOPr/DOPr ligands LP1 and LP2. A description of the development process to discover these dual-target ligands was presented through the synthesis and biological evaluation of compounds with a benzomorphan scaffold. In fact, modifications on *N*-substituent of the benzomorphan-based ligands led to highlighting some requirements proper for the multitarget profile. Moreover, the in vitro and in vivo profiles of LP1 and LP2 are presented. Thus, this review could represent a useful example of the discovery process of new opioid ligands with a safer profile.

## 2. Dual-Target Benzomorphan-Based Ligands LP1 and LP2

Benzomorphan, with morphan, phenylmorphan and phenylpiperidine, originate from morphine structure simplification [34]. The benzomorphan nucleus, aliases 2,6-methano-3-benzazocin-8-ol, provide a rigid backbone for probing the pharmacophoric requirements in opioid receptor interaction, represented by the aromatic ring, the saturated or morphan segment, and the nitrogen substituent. The (−)-(2*R*,6*R*,11*R*) configuration, identical to that of (−)-morphine, is preferable for the interaction with opioid receptors. The (+)-(2*S*,6*S*,11*S*) antipode was able to interact with other targets [35], such as the sigma 1 receptor (σ1R). For example, the synthesis of (+)-(2*S*,6*S*,11*S*)-LP1 analogue [(+)-LP1] gave a compound with a nanomolar σ1R binding affinity (K_i_^σ1^ = 5 ± 0.32 nM) [36].

In the rigid benzomorphan scaffold, the importance of (a) basic nitrogen and (b) phenolic group, at a reciprocal distance comparable to similar functional groups of Tyr^1^ of endogenous opioid peptides, was highlighted [34]. Moreover, basic nitrogen substituents and modifications in the phenolic group are critical for opioid receptor recognition.

Unlike morphine and morphinan analogues, in benzomorphan series the presence of a phenolic group is not crucial for opioid activity. Indeed, in benzomorphan compounds with phenolic group replaced by acetoxy or amine groups, an equivalent or increased antinociceptive effect was described [37,38]. However, the phenolic group replacement with a methyl group or halogens resulted in a reversal of the trend. Also, the *N*-substituent nature is critical in the efficacy profile of the resulting benzomorphan compounds. For instance, an improved opioid receptor interaction was reported as a consequence of methyl substitution with more hydrophobic groups [39]. Otherwise, in morphans and morphinans, a switched agonism to antagonism functional profile was consequent to the introduction of steric hindrance groups, such as *N*-cyclopropylmethyl, -allyl, -dimethyl allyl, -cyclobutylmethyl [40].

In this context, Pasquinucci et al. [41] designed and synthesized a series of benzomorphan derivatives and demonstrated the importance of the nature, size, electronic and steric properties of benzomorphan *N*-substituent in the achievement of a specific functional profile from agonism to antagonism via mixed agonist/antagonist ligands at MOPr, DOPr and KOPr (kappa opioid peptide receptor).

**Figure 2:**
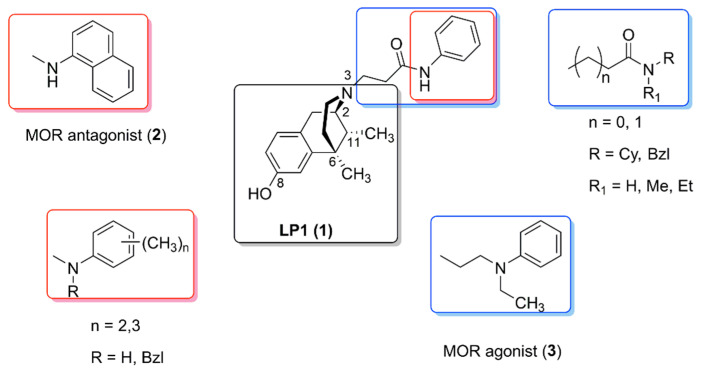
Structures of benzomorphan-based ligands: LP1 and its analogues.

An aromatic ring and/or alkyl residues linked by an *N*-propanamide or *N*-acetamide spacer were first introduced, and an *N*-phenylpropanamide substituent resulted in a potent MOPr agonist/DOPr antagonist ligand (named **LP1**, **1** Figure 2) able to counteract nociceptive pain and behavioral signs of persistent pain with low tolerance-inducing capability [42]. All other amide substituents led to reduced MOPr affinity, and in the *N*-propanamide series the phenyl substitution with a cyclohexyl ring as the increase of the distance between phenyl ring and the benzomorphan core were detrimental [41]. The secondary amide introduction with a higher steric bulk of the amide substituent was also unfavorable. Moreover, shortening of the spacer in the *N*-acetamide series produced a significant loss of MOPr affinity. LP1 also exhibited good DOPr affinity, while all the other compounds had no affinity (Table 1).

Bulkier aromatic groups, such as naphthyl, quinoline and isoquinoline rings, at the *N*-propanamide spacer, were not tolerated and the bulkier size of the *N*-substituent moved the functional profile from agonism to antagonism versus MOPr [43,44]. In particular, in LP1, the phenyl replacement with the 1-naphthyl ring switched to a selective and potent MOPr antagonist (**2**, Figure 2), with a K_i_ of 38 ± 4 nM and an antagonist potency value (pA_2_) of 8.6 in presence of MOPr agonist DAMGO. Moreover, subcutaneously administered, it antagonized the antinociceptive effects of morphine with an AD_50_ of 2.0 mg/kg in a mouse-tail flick test (Table 1).

The LP1 analogue **3** (Figure 2), lacking the amide functionality and with a tertiary *N*-ethylamine group as flexible spacer supporting the phenyl ring, exhibited a selective MOPr agonist profile [45]. In vitro a K_i_^MOR^ of 6.1 nM in a competitive binding assay, and an IC_50_ value of 11.5 nM and an I_max_ of 72% in measurement of the cyclic adenosine monophosphate (*c*AMP) accumulation in human embryonic kidney (HEK293) cells stably expressing MOPr, were detected. In a tail flick test, compound **3** exhibited naloxone-reversed antinociceptive properties with an ED_50_ of 4.33 mg/kg. Thus, the presence of a second positive charge at the *N*-substituent, as well as shorter and more flexible *N*-substituent spacers, addressed the ligand–opioid receptor interaction, mainly at MOPr (Table 1).

Finally, LP1 analogues variously *o*/*m*/*p* polymethylated at the phenyl ring of the *N*- substituent, and analogues featured with tertiary *N*-benzylpropanamide substituents were synthesized (Figure 2) [46]. Biased and unbiased MOPr agonists toward ERK1,2 activity stimulation and on adenylate cyclase inhibition were obtained.

**Figure 3:**
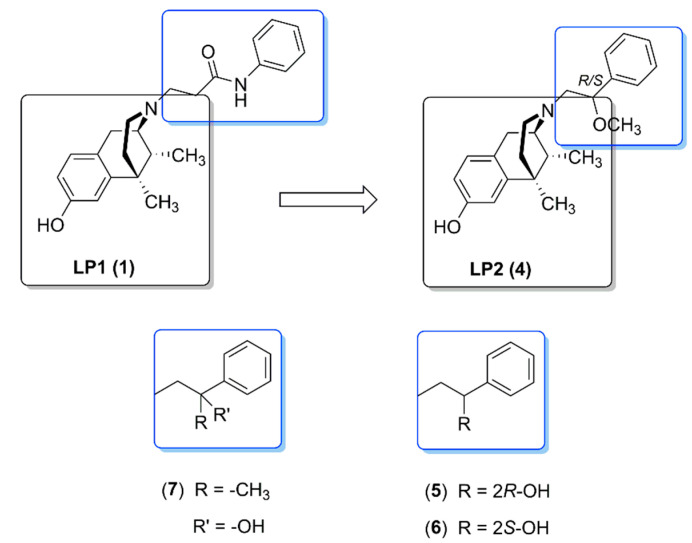
Structures of benzomorphan-based ligands: LP2 and its analogues.

LP1 analogues that maintained the phenyl ring in the *N*-substituent of the benzomorphan scaffold linked to a shorter ethyl spacer bearing a methoxyl (**4**) or hydroxyl (**5**,**6**) or methyl group (**7**) at carbon 2 were synthesized (Figure 3) [47]. In respect to hydroxyl groups, to explore spatial determinants in the proximity of phenyl rings the isomers with chiral (*R*) or (*S*) stereochemistry (**5** and **6**, respectively) were also evaluated [48]. In particular, the introduction of the flexible 2*R*/*S*-methoxyethyl spacer led to a dual-target MOPr agonist/DOPr agonist (named **LP2**, **4**) endowed with a significant long-lasting antinociceptive effect in nociceptive and persistent pain models [47] (Table 1).

In competition binding assays, all synthesized compounds (**4–7**) bounded to the MOPr and KOPr and significantly improved DOPr interactions. In comparison with (*R*) isomer (**6**), the analogue with the (*S*)-2-hydroxy-2-phenylethyl as *N*-substituent (**6**) showed the best MOPr, DOPr and KOPr affinity profile, with K_i_ values of 0.5, 0.8 and 2.22 nM, respectively [48] (Table 1). Thus, the role of the stereocenter in *N*-substituent seems to be important in their binding properties. In GPI and MVD assays, all compounds showed high opioid agonist potency, and their effects were reversed by the selective opioid antagonists naloxonazine (NLX), *nor*-binaltorphimine (*nor*BNI) and NTI [47]. In respect to the lead compound LP1, the *N*-substituent nature of compounds **4**, **5** and **6** shifts the DOPr profile from antagonism to agonism (IC_50_ = 4.4 nM, 10.8 nM and 11.8 nM, respectively). In the mouse-tail flick assay, the antinociceptive effect of compounds **5** and **6** was maximal at 30 min post-administration, and pretreatment with naloxone (3 mg/kg s.c.) fully reversed the agonistic activity. ED_50_ was measured for compound **5** (1.3 mg/kg i.p.) and compound **6** (1.0 mg/kg i.p.) in respect to the ED_50_ value of morphine (2.7 mg/kg s.c.) and LP1 (2.03 mg/kg s.c.). In this series of ligands, the increased flexibility of the *N*-substituent and the presence of a hydroxyl or methoxyl group at carbon 2, as hydrogen bond donor and acceptor, allow an optimal interaction with the opioid binding pocket.

### 2.1. Pharmacological LP1 Fingerprint

#### 2.1.1. In Vitro Biological Evaluation

Radioligand binding assays highlighted the critical importance of the *N*-phenylpropanamide substituent for **LP1** (**1**) affinity and selectivity versus different opioid receptor types [41]. In these in vitro experiments, performed using rat brain membrane and [^3^H]-DAMGO and [^3^H]-DPDPE, high and moderate LP1 affinity for MOPr and DOPr (K_i_^MOPr^ = 0.83 ± 0.05 nM and K_i_^DOPr^ = 29 ± 1 nM) was established.

Knowing the MOPr and DOPr affinity, the functional profile of LP1 was studied measuring the LP1 ability to (a) affect forskolin-stimulated adenylyl cyclase activity in HEK293 cells stably expressing MOPr or DOPr [49] and (b) stimulate [^35^S]GTPγS binding in membranes from HEK293 cells expressing either MOPr or DOPr [42]. The MOPr selective agonist LP1 inhibited, dose-dependently, *c*AMP accumulation in HEK293 cells stably expressing the MOPr. In a similar manner, a dose-dependent inhibition of *c*AMP accumulation in HEK293 cells stably expressing the DOPr treated with LP1 was also detected. However, LP1 was approximately 100-fold less potent than DPDPE at inhibiting *c*AMP accumulation. Similarly, in isolated cell membranes expressing the MOPr, LP1 produced a significant dose-dependent stimulation of [^35^S]GTPγS binding, however displaying an EC_50_ value and maximal efficacy lower than that detected with DAMGO. Similarly, in HEK293 cell membranes expressing the DOPr, LP1 at a concentration of 10 nM resulted in 30% inhibition of [^35^S]GTPγS binding and displayed a weak effect on adenyl cyclase activity. Collectively, these results demonstrate the bifunctional MOPr/DOPr profile of LP1 as assessed both by [^35^S]GTPγS binding and adenylyl cyclase measurements.

The functional profile of LP1 was also verified through ex-vivo experiments on isolated organs [43]. In a GPI assay, LP1 showed a MOPr agonistic profile with a pEC_50_ of 6.1 ± 0.1 comparable to that of DAMGO (pEC_50_ of 6.8 ± 0.03). On the other hand, in the electrically stimulated MVD, LP1 was inactive as an agonist, even at the highest doses. A single concentration (10^−6^ M) of LP1 displaced a rightward shift of the concentration-response curve of DPDPE with a pEC_50_ value of 3.9 ± 0.2, thus producing an antagonistic potency profile with a pA_2_ value of 7.2.

The electrophysiological properties of LP1 (from 1 fM to 100 μM) were also evaluated, using its ability to induce activity changes in primary neuronal cultures from frontal cortex and spinal cord neuronal networks by micro-electrode array (MEA) neurochips [50]. LP1 induced biphasic changes in the general spike and burst activity. LP1 was more potent in the spinal cord (EC_50_^SC1^ = 0.35 fM) than in the frontal cortex (EC_50_^FC1^ = 44 pM). A higher concentration caused a strong activity reduction with an EC_50_^SC2^ = 28 nM and EC_50_^FC2^ = 1 μM. Correlations, by comparing the activity pattern “fingerprint” of LP1 to those of 105 substances in the “NeuroProof GmbH”database, were performed by evaluating the LP1 effects compared with the other compounds, depending on the concentration used. At lower concentrations (10^−15^–10^−10^ M), LP1 revealed an inhibitory effect similar to the MOPr agonist dermorphin characterized by a multitarget MOPr/DOPr profile. Significant similarities to DOPr agonist ligands were not found. These results agree with the MOPr and DOPr multitarget profile LP1.

As all in vivo investigations were performed by s.c. administration of LP1 oxalate salt, due to LP1 freebase low water solubility, another in vitro evaluation was also performed to improve its water solubility [51]. Oxalate salt, in vivo, could cause cytotoxicity such as plasma membrane damage and organelle injury mainly in the kidneys. To evaluate easy formulations for safe administration, the interactions of both oxalate salt and freebase LP1 with a bio-membrane model were investigated. Multilamellar vesicles (MLV) of dimyristoylphosphatidylcholine (DMPC) were used as biological membrane models and potential drug carriers. Using a differential scanning calorimetry (DSC) technique, information about the possible use of an easy delivery system for LP1 freebase was obtained by measuring the dissolution and diffusion in and between the lipidic phases of oxalate salt and freebase LP1. Both compounds were able to interact with the biomembrane model, although the interaction was more prominent for LP1 freebase, which is in agreement with its lipophilicity and non-peptide structure. Coherently, due to the lower water solubility, the aqueous medium did not promote the uptake of LP1 freebase, unlike LP1 oxalate salt. These data demonstrated that LP1 freebase was effectively delivered by the liposomal carrier to a biomembrane model, with a good transfer profile potentially allowing its brain delivery after systemic administration.

#### 2.1.2. In Vivo Pharmacological Evaluation

**Figure 4:**
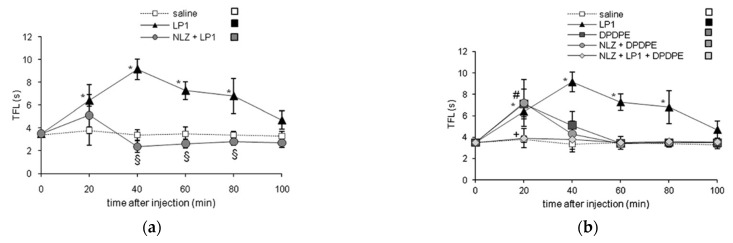
LP1 pharmacological evaluation in tail flick test: (**a**) effect of NLZ (35 mg/kg s.c.) on LP1 (3 mg/kg s.c.) antinociception; (**b**) effect of LP1 (3 mg/kg s.c.) on DPDPE (20 μg/5 μL/rat i.c.v.) antinociception in rats pre-treated with NLZ (35 mg/kg s.c.). Data are expressed by the time-course curve of mean ± S.E.M. tail flick latencies (s). * *p* < 0.05 vs. saline-treated rats. # *p* < 0.05 vs. saline-treated rats. + *p* < 0.05 vs. DPDPE-treated rats. Reproduced with permission from Parenti C. et al. *Life Sci.* **2012**, *90*, 957–61, published by Elsevier.

The interesting in vitro profile of LP1 prompted us to further investigate in vivo its effects in different behavioral pain models. Comparably to morphine (ED_50_ = 2.03 mg/kg s.c.), in tail flick test LP1 elicited a significant antinociceptive effect (ED_50_ = 2.7 mg/kg s.c.) which was completely reversed by NLX [41]. In the same animal pain model, selective MOPr, DOPr and KOPr antagonists confirmed the in vitro results for LP1-induced antinociception [49]. As shown in Figure 4 (panel a), a clear and unequivocal MOPr involvement in the LP1 effect was highlighted. Indeed, the pre-treatment with the selective MOPr antagonist NLZ, at the dose of 35 mg/kg, completely antagonized LP1 antinociception. The DOPr functional profile of LP1 was also confirmed by a tail flick test. In fact, the antinociceptive profile of LP1 was not modified by the selective DOPr antagonist NTI at the dose of 1 mg/kg s.c., whereas LP1 counteracted the antinociception induced by the selective DOPr agonist DPDPE (20 μg/5 μL i.c.v), as shown in Figure 4, panel b. The selective KOPr antagonist *nor*BNI (10 mg/kg s.c.) partially prevented LP1 antinociceptive effects. KOPr involvement in LP1 antinociception was not coherent to binding data, but it could be indirectly related to MOPr activation. In fact, it has been demonstrated that MOPr stimulation induces the release of endogenous dynorphins that act on KOPr to elicit antinociception [52,53]. These results confirm the LP1 dual-target profile i.e., to act as an MOP agonist/DOPr antagonist.

**Figure 5:**
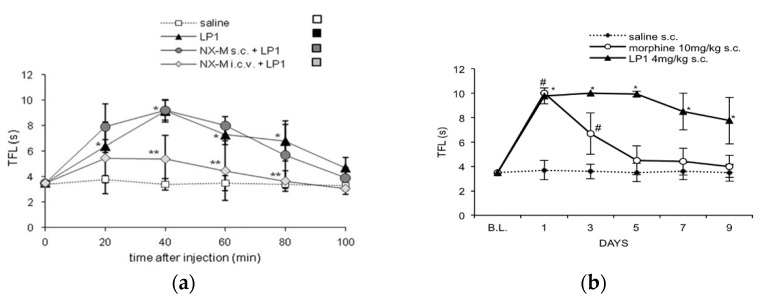
LP1 pharmacological evaluation in tail flick test: (**a**) effect of NX-M s.c. (3 mg/kg) or i.c.v. (5 μg/5 μL/rat) administered, on LP1 (3 mg/kg s.c.) antinociception. Data are expressed by the time-course curve of mean ± S.E.M. tail flick latencies (s) * *p* < 0.05 vs. saline-treated rats. ** *p* < 0.05 vs. LP1-treated rats; (**b**) effects of morphine (○) (*n* = 8) and LP1 (▲) (*n* = 10) on development of antinociceptive tolerance. Data are expressed as the mean ± S.D. * *p* < 0.05 vs. saline-treated rats (*n* = 8); # *p* < 0.05 vs. saline-treated rats (*n* = 8). Reproduced with permission from Parenti C. et al. *Life Sci.* **2012**, *90*, 957–61 (panel a) and Pasquinucci L. et al. *Bioorg. Med. Chem.* **2010**, *18*, 4975–82 (panel b), published by Elsevier.

The LP1 antinociceptive effect was also determined in naloxone methiodide (NX-M) pre-treated rats [49]. A significant reduction of LP1 antinociceptive effect was reported after i.c.v. administration of NX-M (5 μg/5 μL), a quaternary derivative that does not readily cross the blood–brain barrier (BBB), whereas the s.c. injection of NX-M (3 mg/kg) did not affect tail flick latencies (Figure 5, panel a). Thus, these data demonstrated that the LP1 compound exerts its action predominantly in the central nervous system (CNS).

LP1 tolerance-inducing capability was defined by tail flick test in comparison to morphine [42]. A significant loss of antinociceptive effect was detected on the third day of treated rats with morphine injection (10 mg/kg, s.c. twice a day), while LP1, administered at the dose of 4 mg/kg s.c. with the same experimental protocol, maintained its antinociceptive profile until day 9 (Figure 5, panel b). Thus, LP1 produced the same morphine antinociceptive effect with a less pronounced development of tolerance.

**Figure 6:**
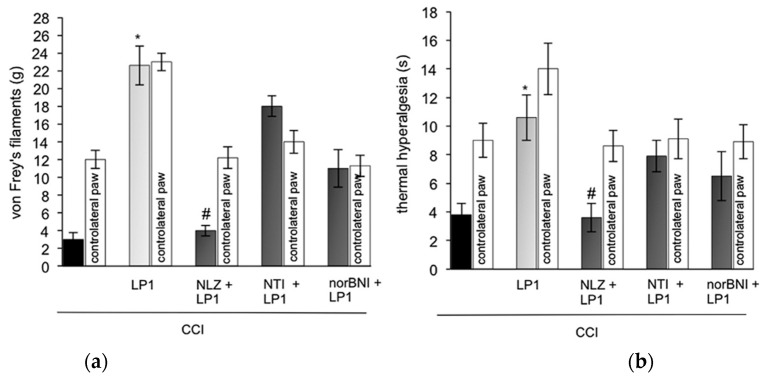
LP1 pharmacological evaluation in CCI rats: Antagonistic effect of NLZ (35 mg/kg s.c.), NTI (1 mg/kg s.c.) and *nor*BNI (10 mg/kg s.c.) on the antiallodynic (**a**) and antihyperalgesic (**b**) effects of LP1 (3 mg/kg s.c.) in CCI rats. Graphs show values at 45 min on day 14 for mechanical allodynia, measured with von Frey’s filaments, and day 8 for thermal hyperalgesia, measured with Plantar test. Results are expressed in grams(g) (mechanical allodynia) or in seconds(s) (thermal hyperalgesia) and represent the mean ± S.E.M. (8–10 rats). * *p* < 0.05 vs. CCI vehicle treated-rats; # *p* < 0.05 vs. LP1-treated rats. Reproduced with permission from Parenti C. et al. *Neuropharmacology* **2013**, *71*, 70–82, published by Elsevier.

The low tolerance-inducing capability of LP1 prompted its evaluation in animal models of persistent pain, such as neuropathic and inflammatory pain [50]. In chronic constriction injury (CCI) rats, at the 14th day after surgery when allodynic thresholds decreased, LP1, s.c., produced a significant antiallodynic effect (ED_50_ = 3.2 mg/kg at 45 min). The maximal effect was recorded at 45 min and 60 min after treatment. In the same animal model, at day 8, when hyperalgesic thresholds decreased, LP1 caused a significant anti-hyperalgesic effect in response to a radiant heat stimulus with plantar test (ED_50_ = 2.7 mg/kg at 45 min). To evaluate the contribution of opioid receptor types in LP1-induced antiallodynic and antihyperalgesic effects, the multitarget LP1 profile was assessed using selective opioid antagonists (Figure 6, panel a and b, respectively). The selective MOPr antagonist NLZ completely antagonized LP1 anti-allodynic and antihyperalgesic effects. In contrast, 1 mg/kg s.c. of the selective DOPr antagonist NTI did not alter the LP1 effects, and the selective KOPr antagonist *nor*BNI only slightly blocked the antiallodynic and antihyperalgesic effects of LP1.

**Figure 7:**
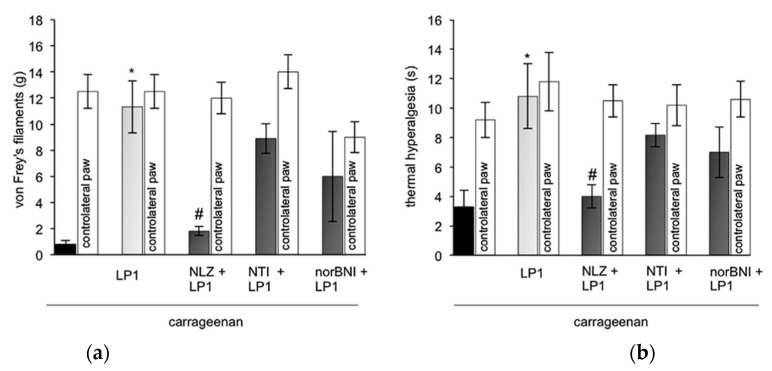
LP1 pharmacological evaluation in carrageenan edema assay: antagonistic effect of NLZ (35 mg/kg s.c.), NTI (1 mg/kg s.c.) and *nor*BNI (10 mg/kg s.c.) on the antiallodynic (**a**) and antihyperalgesic (**b**) effect of LP1 (3 mg/kg s.c.) injected 15 min prior to i.pl. carrageenan (2%/0.1 mL/rat). Graphs show values at 3 h. Mechanical and thermal thresholds were measured with von Frey’s filaments and Plantar test, respectively. Results are expressed in grams(g) (mechanical allodynia) or in seconds(s) (thermal hyperalgesia) and represent the mean ± S.E.M. * *p* < 0.05 vs. carrageenan treated-rats; # *p* < 0.05 vs. LP1-treated rats. Reproduced with permission from Parenti C. et al. *Neuropharmacology* **2013**, *71*, 70–82, published by Elsevier.

In an analogous manner, LP1 s.c. administered in the left paw, significantly reduced allodynic (ED_50_ = 2.8 mg/kg) and hyperalgesic (ED_50_ = 3.1 mg/kg) thresholds in a model of inflammatory pain induced by carrageenan [50]. The antinociceptive effects of the multitarget LP1 profile was assessed using selective opioid antagonists, (Figure 7, panel a and b, respectively). As in CCI rats, NLZ completely reversed LP1 antiallodynic and antihyperalgesic effects, while the selective DOPr antagonist NTI did not modify the LP1 effect, and *nor*BNI partially blocked its antiallodynic and antihyperalgesic effects.

**Figure 8:**
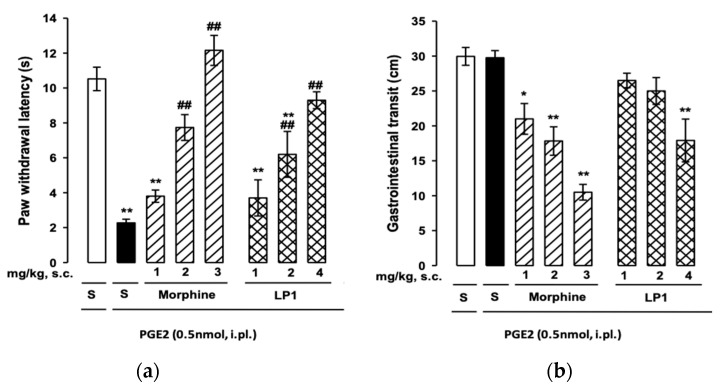
(**a**) Effects of morphine, LP1 or saline (S) on PGE2-induced heat hyperalgesia: statistically significant differences between the values obtained in mice i.pl. injected with saline and PGE2: * *p* < 0.05, ** *p*  <  0.01, and between the values obtained in mice treated with PGE2 alone or associated with morphine or LP1: ## *p*  <  0.01; (**b**) effects of morphine, LP1 or saline (S) on gastrointestinal transit: statistically significant differences between the values obtained in saline-treated group and mice treated with morphine or LP1: * *p* < 0.05, ** *p*  <  0.01.

Moreover, the LP1 anti-hyperalgesic effect was also rescued through mouse PGE2-induced heat hyperalgesia and compared to morphine (Figure 8, panel a) [46]. In agreement with previous findings, both morphine (1–3 mg/kg, s.c.) and LP1 (1–4 mg/kg, s.c.) induced a dose-dependent increase in paw withdrawal latency in PGE2-treated mice, reaching values similar to those detected in control animals.

As constipation is known as an opioid-induced side effect [54], LP1 effects on gastrointestinal transit were also tested (Figure 8, panel b) [46]. Immediately after the evaluation of the behavioral responses to heat stimulus, mice received intragastrically an activated charcoal solution. Morphine, at a dose without antinociceptive effect (1 mg/kg), already inhibited intestinal transit, and this effect dose-dependently increased up to the highest dose tested (3 mg/kg). In contrast, LP1 inhibited gastrointestinal transit only at the highest dose tested (4 mg/kg), which induced a maximal antihyperalgesic effect. These results indicated that the MOPr agonist/DOPr antagonist LP1 is an effective compound in both nociceptive and persistent pain models, and displays a more favorable and safe profile lacking the adverse effects of morphine.

### 2.2. Pharmacological LP2 Evaluation

#### 2.2.1. In Vitro Biological Evaluation

LP2 came about through structure–activity relationship (SAR) studies performed on LP1. Analogously to LP1, its efficacy profile was in vitro defined [47]. Firstly, the opioid receptor subtype affinity profile of the benzomorphan-based compound LP2 was evaluated by competitive radioligand binding assays (Table 1). LP2 retained the MOPr affinity of the lead compound LP1 (K_i_^MOPr^ = 1.08 nM *versus* K_i_^MOPr^ = 0.83 nM, respectively) while its affinity for DOPr was increased. In fact, the DOPr K_i_ value of LP2 was four times lower than the K_i_ value of LP1 (K_i_^DOPr^ = 6.6 nM vs. K_i_^DOPr^ = 29.1 nM, respectively). LP2 differs from LP1 by the nature and chiral feature of the *N*-substituent. In LP2, the lack of the amide group in its *N*-substituent is well tolerated by MOPr and significantly improved DOPr interaction. Moreover, in comparison to LP1, the increased flexibility of the *N*-substituent could allow an optimal interaction with the DOPr binding pocket.

The LP2 functional profile was determined in the presence and absence of a fixed concentration of selective MOPr (NLX), DOPr (NTI) and KOPr (*nor*BNI) antagonists ex vivo by GPI and MVD assays. Initially, LP2 showed high opioid agonist potency in GPI (IC_50_ = 21.5 ± 1.3 nM) and MVD (IC_50_ = 4.4 ± 0.7 nM) assays. Indeed, increasing concentrations of LP2 produced a significant and concentration-dependent inhibition in electrically stimulated GPI and MVD measurements. LP2 retained a significative MOPr potency, although it resulted in an eleven times lower effect in comparison to LP1 (IC_50_ = 1.9 ± 0.6 nM). Significantly, in respect to the lead compound LP1, the *N*-substituent nature of LP2 shifted the DOPr profile from antagonism to agonism. Thus, LP2 resulted as a dual-target MOPr/DOPr agonist, unlike LP1 that was a MOPr agonist/DOPr antagonist.

The DOPr agonist profile of LP2 could be important for its evaluation in chronic pain animal models, considering that DOPr agonists were reported to be able to induce potent antihyperalgesics properties in these assays. Unfortunately, some agonists, such as SNC80 and (+)BW373U86, have pro-convulsant properties that have limited their clinical development. However, literature data described these pro-convulsant properties as “ligand specific” [55,56], and therefore the development of a DOPr agonist without these adverse effects is nowadays a valuable approach [57,58]. Moreover, G-protein biased opioid agonists were identified, and the potential relationship between the in vitro bias profile and in vivo antinociception and side effects was hypothesized [59]. The development of biased agonists able to target specific signaling pathways was pursued, and G protein-biased DOPr agonists seems to be an approach to develop ligands effective in chronic pain states with a reduced adverse effect profile. Literature data suggested that receptor internalization is mediated by arrestin activation, and low-internalizing agonists showed a decreased tendency to induce convulsions. For example, DOPr agonists such as PN6047, with limited arrestin signaling, was identified as a useful compound for the treatment of chronic pain, without displaying proconvulsant activity or other opioid-mediated adverse effects [60].

Thus, the functional activity of LP2 was further investigated using a BRET assay [48] to evaluate LP2 ability to promote receptor/G-protein or receptor/β-arrestin 2 interactions. For that reason, concentration-response experiments to evaluate receptor/G-protein interaction were performed in membrane extracts from SH-SY5Y cells stably co-expressing the MOPr or DOPr/RLuc and Gb1/RGFP fusion proteins. To evaluate receptor/β-arrestin 2 interactions, SH-SY5Y cells stably expressing the MOPr or DOPr/RLuc and the β-arrestin 2/RGFP fusions were used. The intrinsic activity of the tested compound was evaluated as a fraction of the DADLE maximal-stimulated BRET ratio (α = 1).

LP2 promoted MOPr/G-protein interactions and mimicked the maximal effect of DADLE (pEC_50_ = 6.89), being 10 times more potent with a pEC_50_ of 7.90. LP2 stimulated the MOPr/β-arrestin 2 interaction, mimicking the stimulatory response of DADLE with slightly lower efficacy but with a 5 times higher potency. Thus, LP2 displayed a modest (<10 times) bias toward the G-protein. In parallel experiments, LP2 behaved as a full DOPr agonist (pEC_50_ = 7.02) with similar potency and efficacy as DADLE (pEC_50_ = 7.23). Moreover, in DOPr/β-arrestin 2 interactions, LP2 mimicked the maximal effects of DADLE (pEC_50_ = 7.69 and pEC_50_ = 5.65, respectively) being, however, less potent. Thus, in DOPr LP2 showed a statistically significant and large (200-times) bias toward the G-protein. Thus, the LP2 dual target MOPr/DOPr agonist with a biased profile could provide a safer treatment opportunity.

#### 2.2.2. In Vivo Pharmacological Evaluation

The dual target MOPr/DOPr agonist LP2 was also evaluated in vivo by tail-flick test in mice [47]. LP2 produced a dose-dependent antinociceptive effect, and the maximal antinociceptive threshold was reached between 45 and 60 min after injection and lasted until 180 min after treatment. Compared to morphine and LP1, LP2 evoked a potent antinociceptive effect with an ED_50_ of 0.9 mg/kg i.p. In contrast to morphine, whose antinociceptive effect started to decline 60 min post-administration, LP2 showed a longer lasting antinociceptive effect, measured also at 180 min after administration. NLX (3 mg/kg s.c.) pretreatment prevented the induced antinociceptive LP2-effect. Thus, our in vivo evaluation was consistent with the in vitro data and suggested that the LP2 antinociceptive effect is mediated by the MOPr and DOPr, and that it is more potent than morphine and LP1.

In addition, LP2, for its pharmacodynamic profile and in vivo effect in nociceptive pain, was investigated in a model of inflammatory pain sustained by mechanisms of central sensitization, i.e., the mouse formalin test [61]. This test involves the i.pl. injection of a dilute solution of formalin (5%, 10 μL) that induces a biphasic response reflecting two different forms of pain. A rapid pain response is induced immediately after formalin injection that lasts 10 min (phase I) and represents a form of acute pain due to the direct activation of nociceptors. In addition, a late pain response to formalin injection starts after 10–15 min and lasts until 50–60 min from the injection of formalin (phase II). This second phase of pain behavior is considered more relevant from a clinical point of view, because it represents a form of more persistent, tonic, inflammatory pain sustained by the development of mechanisms of nociceptive sensitization.

LP2, i.p. administered at the dose of 0.5, 0.65, 0.75, 0.85, 1 mg/kg 15 min before formalin injection, dose-dependently reduced both phases of the mouse formalin test with an ED_50_ value of 0.88 mg/kg and 0.79 mg/kg in phase I and phase II, respectively.

**Figure 9:**
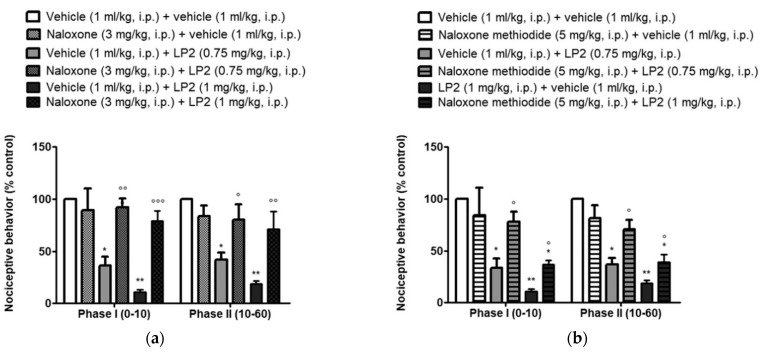
LP2 pharmacological evaluation in formalin test: (**a**) the antinociceptive effect of LP2 is blocked by NLX pretreatment. Results are mean ± S.E.M. (*n* = 6–8 per group) * *p* < 0.05; * *p* < 0.01 vs. vehicle, ° *p* < 0.05; °° *p* < 0.01; °°° *p* < 0.001 vs. LP2; (**b**) the antinociceptive effect of LP2 is partially blocked by NX-M pretreatment. Results are mean ± S.E.M. (*n* = 6–8 per group) * *p* < 0.05; ** *p* < 0.01 vs. vehicle, ° *p* < 0.05 vs. LP2. Reproduced with permission from Pasquinucci L. et al. *Eur. J. Pharmacol.* **2019**, *847*, 97–102, published by Elsevier.

To evaluate the systemic effect of LP2, the non-selective opioid receptor antagonist NLX (3 mg/kg, i.p.) was administered. It significantly antagonized the antinociceptive effects of LP2 in both phases of the mouse formalin test (Figure 9, panel a). Mice pretreatment with NX-M allowed to evaluate a possible antinociceptive LP2 effect outside CNS. NX-M (5 mg/kg, i.p.) indeed antagonized the LP2 antinociceptive effect at a lower dose of 0.75 mg/kg, but not that at the dose of 1.0 mg/kg, suggesting that the LP2 antinociceptive effect is partially mediated by peripheral opioid receptors (Figure 9, panel b). Collectively, these results suggest that LP2 is effective in the formalin test and that its antinociceptive effects were mediated both at central and peripheral levels.

**Figure 10:**
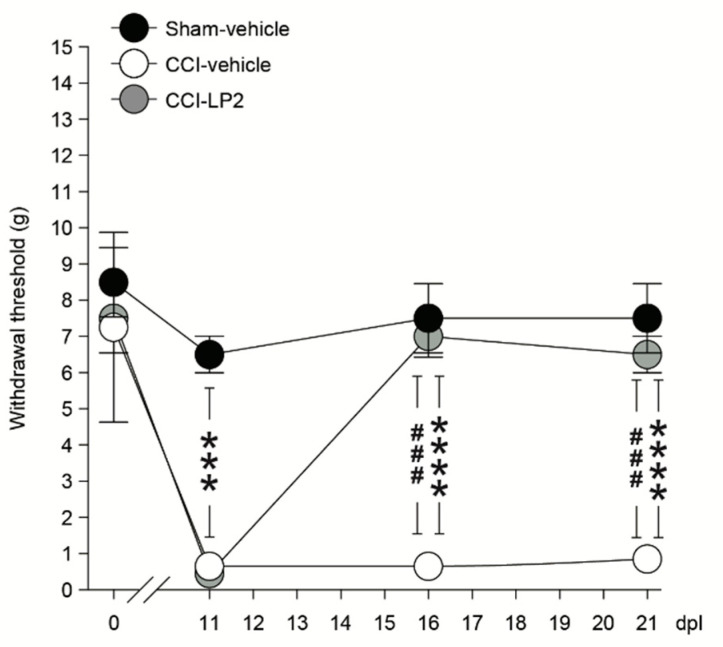
Withdrawal thresholds measured with von Frey’s filaments on sham-vehicle, CCI-vehicle rats and CCI-LP2-treated rats at 0, 11, 16, and 21 dpl. Data are shown as mean ± SEM of *n* = 4 rats per group. *** *p* value < 0.001, **** *p* value < 0.0001 vs. sham-vehicle, and ^###^ *p* value < 0.001 vs. CCI-vehicle. Reproduced with permission from Vicario N. et al. *Mol. Neurobiology* **2019**, *56*, 7338–7354, published by Springer, 2019.

The effects of LP2 in the experimental model of neuropathic pain induced by the unilateral sciatic nerve CCI on male Sprague–Dawley rats was also evaluated [62]. Rats received a daily i.p. injection of 0.9 mg/kg of LP2, starting at 11 until 21 days post-ligatures (dpl). A significant increase of the allodynic withdrawal threshold in CCI rats as compared to CCI-vehicle rats was determined from 11 up to 21 days post ligature (dpl) (Figure 10). Moreover, a repeated LP2 administration seems to maintain antinociceptive effects.

**Figure 11:**
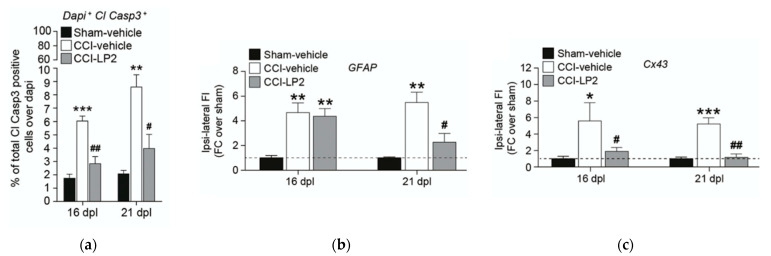
(**a**) LP2 reduces cleaved caspase 3-positive neurons in ipsi-lateral dorsal horns of CCI rats: quantification of Cl Casp3 and DAPI (4′,6-diamidino-2-phenylindole, dihydrochloride) double cells over DAPI in ipsi-lateral dorsal horns of sham-vehicle, CCI-vehicle, and CCI-LP2-treated rats at 16 and 21 dpl. Data are shown as mean percentage ± SEM of n = 3 rats per group. Dpl, days post-ligatures; ** *p* value < 0.01 and *** *p* value < 0.001 vs. sham-vehicle and # *p* value < 0.05 and ## *p* value < 0.01 vs. CCI-vehicle. MOPr and DOPr targeting with LP2 reduces GFAP and Cx43 levels in ipsi-lateral dorsal horns of CCI rats: quantification of GFAP (**b**) and Cx43 (**c**) fluorescence intensity in ipsi-lateral dorsal horns of sham-vehicle, CCI-vehicle, and CCI-LP2- treated rats. Data are shown as mean FC over sham-vehicle ± SEM of n = 4 rats per group. Dpl, days post-ligatures; FI, fluorescence intensity; FC, fold change. * *p* value < 0.05, ** *p* value < 0.01, and *** *p* value < 0.001 vs. sham-vehicle. # *p* value < 0.05 and ## *p* value < 0.01 vs. CCI-vehicle. Reproduced with permission from Vicario N. et al. *Mol. Neurobiology* **2019**, *56*, 7338–7354, published by Springer, 2019.

The effects of LP2 treatment in the spinal cord of ipsi- and contra-lateral dorsal horns were also evaluated during the time-course of neuropathic pain [62]. The total number of Cl Casp3-positive cells were reduced in ipsi-lateral dorsal horn at 16 dpl (2.8 ± 0.5% CCI-LP2 vs. 6.0 ± 0.4% CCI-vehicle) and at 21 dpl. (4.0 ± 1.1% CCI-LP2 vs. 8.6 ± 0.9% CCI-vehicle), confirming the neuroprotective effects of LP2 treatment during CCI-induced neuropathy (Figure 11, panel a). Pro-apoptotic signaling in ipsi-lateral dorsal laminae exert pro-gliosis effects on spinal cord astrocytes in ipsi-lateral dorsal horns [61]. During the time-course of neuropathic pain, both GFAP and Cx43 levels were increased from the early phases of chronic pain [63], suggesting that astroglial communication mediated by Cx43 plays a key role in promoting pain chronicization during neuropathies. Dual-target opioid ligand LP2 reverted Cx43 levels earlier than GFAP astrogliosis, which was still ongoing after 5 days of treatment and significantly reduced only at 21 dpl (Figure 11, panel b,c). Thus, in this pathological condition, LP2-mediated Cx43 reduction seems to affect reactive astrogliosis in neuropathies. Immunofluorescence analysis on spinal cord sections revealed reactive Cx43 activation in ipsi-lateral dorsal horns of CCI-vehicle rats with high Cx43-GFAP co-localization profiles, which was significantly reverted by LP2 treatment at 21 dpl. These data confirmed a significant and long-lasting modulation of astrogliosis, mediated by the MOPr/DOPr ligand LP2.

## 3. Conclusions

There is an unmet clinical need to develop novel pharmaceuticals for persistent pain. Our data showed that the novel dual targeting of MOPr/DOPr benzomorphan-based ligands LP1 and LP2 could represent an example of a “context-dependent antinociceptive drug”. Opioids that combine MOPr agonist–DOPr antagonist activity or MOPr/DOPr agonist activity may be effective antinociceptive agents that are able to attenuate MOPr-mediated side effects. Both LP1 and LP2 have been shown to be effective in animal models of inflammatory and neuropathic pain, and LP1 showed a safer profile of side effects.

In addition, MOPr/DOPr agonist profile of LP2 could be useful in persistent neuropathic pain conditions associated with several comorbidities, such as depression. In fact, the effectiveness of DOPr agonists in models of anxiety and depression was established [60,64]. Notably, we recently demonstrated a role of LP2 vs. TGF-β involved in depression [65].

## Figures and Tables

**Table 1:** In vitro data and tail flick test of benzomorphan-based ligands.

Compd	K_i_ (nM) ± S.D.			IC_50_ (nM) ± S.D.		ED_50_ mg/kg
				GPI ^e^	MVD ^e^	Tail Flick Test
	**MOPr**	**DOPr**	**KOPr**	**MOPr**	**DOPr**	
LP1(**1**) ^a^	0.83 ± 0.05	29.1 ± 1	110 ± 6	6.8 ± 0.03 ^b^	pA_2_ of 7.2	2.03
(**2**) ^b^	38 ± 4	210 ± 30	800 ± 160	pA_2_ of 8.6	-	AD_50_ 2.0
				**IC_50_ (nM) ± S.D.**		
				**cAMP inhibition**		
(**3**) ^c^	6.1 ± 0.5	147 ± 5.7	31 ± 1.3	11.5 ± 2.5	-	4.33
				**IC_50_ (nM) ± S.D.**		
				**GPI ^e^**	**MVD ^e^**	
LP2 (**4**) ^d^	1.08 ± 0.10	6.60 ± 0.60	15.22 ± 0.80	21.5 ± 1.3	4.4 ± 0.7	0.9
(**5**) ^d^	2.47 ± 0.3	9.6 ± 0.5	7.30 ± 0.2	49.2 ± 2	10.8 ± 1.3	1.3
(**6**) ^d^	0.5 ± 0.2	0.8 ± 0.2	2.22 ± 0.2	9.9 ± 0.9	11.8 ± 1	1.0
(**7**) ^d^	60 ± 1.3	160 ± 2.8	10.51 ± 0.9	194.4 ± 33	793.5 ± 43	-

^a^ [41]; ^e^ [42]; ^b^ [43]; ^c^ [45]; ^d^ [47]; GPI guinea pig ileum, MVD mouse vas deferens. Data reproduced with permission from ^a^ Copyright 2010, Elsevier, ^e^ Copyright 2012, Elsevier, ^b^ Copyright 2016, Elsevier, ^d^ Copyright 2017, Elsevier.

## Data Availability

The data presented in this study are available on request from the corresponding author.

## Sample Availability

Samples of the compounds LP1 and LP2 are available from the authors.
